# Crystal structure of potassium tri­ethyl­hydridoborate (‘superhydride’)

**DOI:** 10.1107/S2056989021004734

**Published:** 2021-05-07

**Authors:** Ann Christin Fecker, Matthias Freytag, Marc D. Walter, Peter G. Jones

**Affiliations:** a Institut für Anorganische und Analytische Chemie, Technische Universität Braunschweig, Hagenring 30, D-38106 Braunschweig, Germany

**Keywords:** crystal structure, potassium, hydridoborate, superhydride

## Abstract

In the title compound, formally K^+^·C_6_H_16_B^−^, the contact sphere of potassium consists of eleven hydrogen atoms from three different anions, assuming an arbitrary cut-off of 3 Å. The shortest inter­action, 2.53 (2) Å, involves the hydridic hydrogen H01, which fulfils a bridging function in the formation of chains of KHBEt_3_ units parallel to the *a* axis [K1—H01^i^ 2.71 (2) Å, K1—H01—K1^ii^ 126.7 (9)°, operators *x*∓1/2, −*y* + , −*z* + 1].

## Abstract 2

The structure of KHBEt_3_ is polymeric, involving chains linked by K—H—K motifs *via* the hydridic hydrogen.

## Chemical context   

The title compound KHBEt_3_ was first prepared by Ziegler and Lehmkuhl from NaBEt_3_H and potassium amalgam (Ziegler & Lehmkuhl, 1963 ▸), but a more convenient approach was reported a few years later using KH and BEt_3_ in toluene (Binger *et al.*, 1968 ▸). Alternatively, the latter reaction may also be performed in THF (Brown & Krishnamurthy, 1978 ▸). Since its original synthesis this so-called ‘superhydride’ reagent has found widespread applications, *e.g.* as a reducing reagent in organic synthesis (Brown & Hubbard, 1979 ▸; Ito *et al.*, 1985 ▸; Yoon *et al.* 1987 ▸, 1989 ▸), for the generation of low-valent transition-metal complexes (Bönnemann & Korall, 1992 ▸), and as a hydride transfer reagent resulting in well-defined metal–hydride complexes (Smith *et al.*, 2003 ▸; Pfirrmann *et al.*, 2008 ▸; Walter *et al.*, 2011 ▸; Maekawa *et al.*, 2012 ▸). Despite it being a reagent in frequent use, the structure of KHBEt_3_ has so far remained elusive. The few reported examples of structures containing KHBEt_3_ include its adducts with polydentate amines such as *N*,*N*,*N*′,*N*′-tetra­methyl­ethylenedi­amine (TMEDA) and *N*,*N*,*N*′,*N*",*N*"-penta­methyl­diethylenetri­amine (PMDETA) (Haywood & Wheatley, 2009 ▸). During our study on the coordination chemistry of enanti­omerically pure constrained-geometry complexes of the rare-earth metals bearing a dianionic *N*-donor functionalized penta­dienyl ligand, we accidentally obtained crystals of solvent-free KHBEt_3_ unsupported by any further ligands (see *Synthesis and crystallization*) and here report its structure.
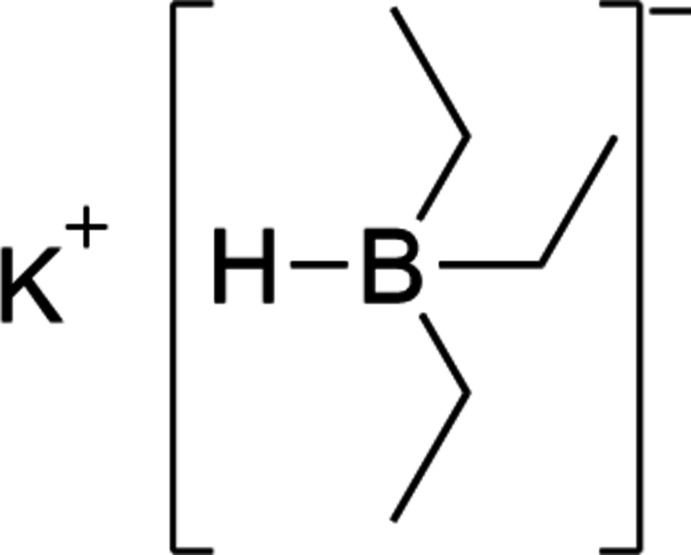


## Structural commentary   

**Figure 1:**
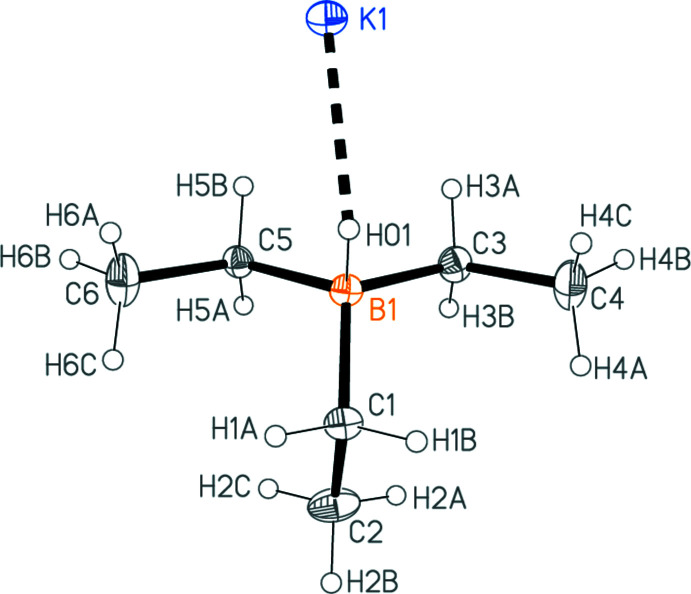
The asymmetric unit of KHBEt_3_. Ellipsoids are drawn at the 50% level. Only the shortest K1—H contact is drawn explicitly.

**Figure 2:**
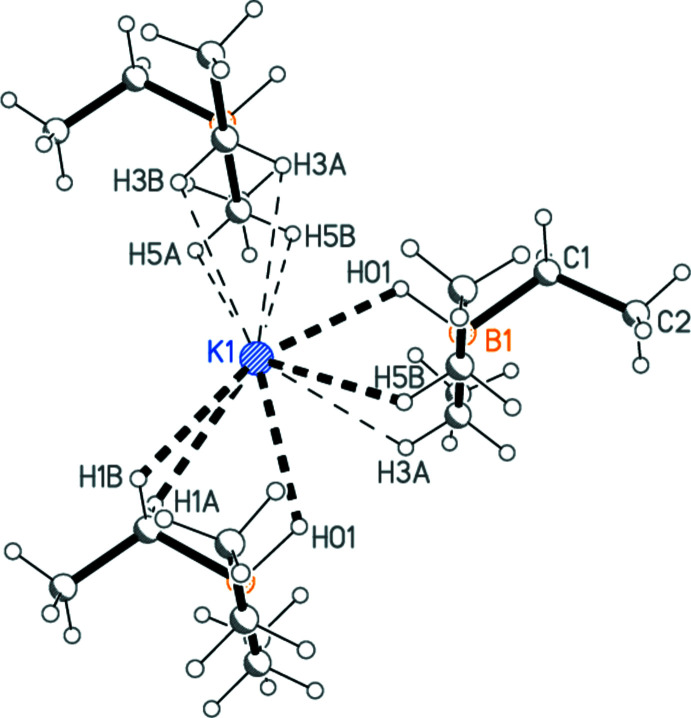
The environment of the potassium atom in KHBEt_3_, showing ten of the eleven K—H contacts < 3 Å to three neighbouring hydridotri­ethyl­borate units. Radii are arbitrary. K—H distances shorter than 2.8 Å are shown as thick dashed bonds, whereas those greater than 2.9 Å are shown as thin dashed bonds. The anion on the right corresponds to the asymmetric unit; the anions at top and bottom were generated by the operators 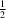 + *x*, 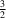 − *y*, 1 − *z* and −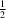 + *x*, 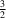 − *y*, 1 − *z*, respectively. The contact to H2*B* of a fourth anion (at 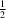 − *x*, 1 − *y*, −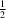 + *z*) is omitted for clarity.

The asymmetric unit of KHBEt_3_ is shown in Fig. 1 ▸. Selected inter­atomic distances and angles are shown in Table 1 ▸. The shortest contact involving the potassium atom is K1—H01 at 2.53 (2) Å, but K1—H5*B* (not drawn explicitly) is not much longer at 2.69 Å. If the neighbouring asymmetric units generated by the 2_1_ screw axis parallel to the *a* axis (see next section) are considered, there are a total of eleven K1—H distances shorter than 3 Å, with no clear limit as to what might be considered a ‘bonding’ distance. One further such distance involves the 2_1_ screw axis parallel to the *c* axis. The environment of the potassium atom is shown in Fig. 2 ▸. For comparison, one may note the K—H distance of 2.85 Å in potassium hydride (Kuznetsov & Shkrabkina, 1962 ▸), which, however, is regarded as an essentially ionic compound, crystallizing in the NaCl lattice type with coordination number 6 (*cf*. the ionic formulation of the title compound in Table 2 ▸, which is certainly a considerable oversimplification). Some K⋯H contacts of *ca* 2.8–2.9 Å, involving methyl hydrogen atoms, have been postulated as structurally significant in a TMEDA complex of potassium diiso­propyl­amide (Clegg *et al.*, 1998 ▸). Similarly, the distances from K1 to carbon and boron atoms range upwards from 3.103 (2) and 3.205 (2) Å, respectively. The bonding to CH_*n*_ and BH moieties may involve multi-centre inter­actions, but we do not wish to speculate on their exact nature. The coordination geometry at the boron atom is as expected tetra­hedral to a good approximation.

## Supra­molecular features   

**Figure 3:**
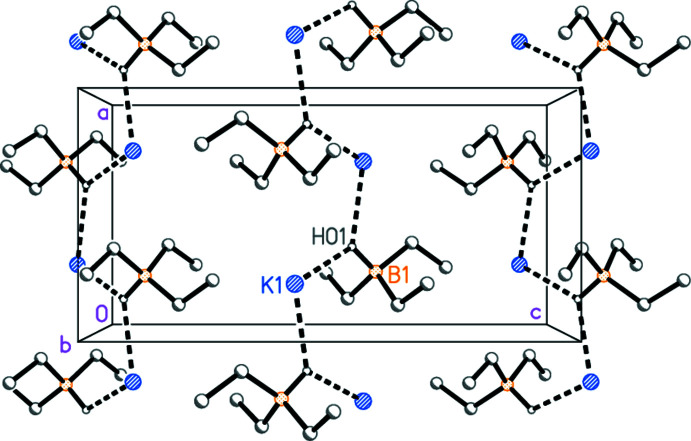
Simplified packing diagram of KHBEt_3_ viewed parallel to the *b* axis. Hydrogen atoms except for H01 are omitted.

To a first approximation, ignoring all inter­actions at K1 except for K1—H01, the mol­ecules are connected by the appropriate 2_1_ operators to form chains parallel to the *a* axis (Fig. 3 ▸). The hydridic hydrogen atom acts as the main bridging group, with K1—H01^i^ = 2.71 (2) Å, H01—K1—H01^i^ = 104.0 (4)°, K1—H01—K1^ii^ = 126.7 (9)°. The distance between adjacent potassium atoms in the chain is 4.6839 (6) Å.

## Database survey   

A CSD search with *ConQuest* (Bruno *et al.*, 2002 ▸) for organic hydridoborate derivatives involving K—H bonds led to the above-mentioned complexes [K(TMEDA)Et_3_BH]_2_ and [K(PMDETA)Et_3_BH]_2_ (Haywood & Wheatley, 2009 ▸, refcodes CUNNEF and CUNNIJ) with K—H distances of 2.52, 2.58 (3) and 2.64, 2.69 (3) Å, respectively, in the central K_2_H_2_ rings. A similar structure (refcode OZAZAR), but with 1,3,5-tri­methyl-1,3,5-tri­aza­nonane, was reported by Krieck *et al.* (2010 ▸), with K—H = 2.56, 2.59 (3) Å. Somewhat more complex structures, involving cyclic boranes and additional aromatic ligands at the potassium atom, have been reported by Grigsby & Power (1996 ▸; refcode TIZYAC, K—H = 2.54, 2.68 Å) and Chen *et al.* [2007 ▸; refcode MITWUI, K—H = 2.65–2.92 (1) Å].

## Synthesis and crystallization   

We attempted the preparation of a rare-earth metal hydride by salt metathesis between [{(η^5^:κ-*N*-pdl*SiMe_2_NtBu)La(thf)}_2_(μ-Cl)] (Jones *et al.*, 2021 ▸) and 2 equiv. of KHBEt_3_ (1 *M* in THF) in *n*-hexane. The standard work-up procedure included removal of the solvent under dynamic vacuum, extraction of the residue with *n*-hexane and filtration. The filtrate was concentrated and cooled to 243 K. After several days, a few pale-yellow crystals were harvested. However, in contrast to our expectations, these did not consist of [{(η^5^:κ-*N*-pdl*SiMe_2_NtBu)La(thf)}_2_(μ-H)], but of the starting reagent KHBEt_3_.

## Refinement   

Crystal data, data collection and structure refinement details are summarized in Table 2 ▸. The BH hydrogen atom was refined freely. The methyl groups were refined as idealized rigid groups allowed to rotate but not tip (AFIX 137; C—H = 0.98 Å, H—C—H = 109.5 °). The methyl­ene hydrogens were included using a riding model starting from calculated positions (C—H = 0.99 Å). The *U*_iso_(H) values were fixed at 1.2 (for methyl­ene groups) or 1.5 (for methyl groups) times the equivalent *U*_eq_ value of the parent carbon atoms.

## Supplementary Material

Crystal structure: contains datablock(s) I, global. DOI: 10.1107/S2056989021004734/yz2007sup1.cif

Structure factors: contains datablock(s) I. DOI: 10.1107/S2056989021004734/yz2007Isup2.hkl

CCDC reference: 2081809

Additional supporting information:  crystallographic information; 3D view; checkCIF report

## Figures and Tables

**Table 1:** Selected geometric parameters (Å, °)

K1—C1^i^	3.103 (2)	K1—H2*B* ^iii^	2.83
K1—B1	3.205 (2)	K1—H3*B* ^ii^	2.93
K1—C5	3.310 (2)	K1—H3*A*	2.93
K1—C3^ii^	3.387 (2)	K1—H5*A* ^ii^	2.94
K1—C5^ii^	3.396 (2)	K1—H3*A* ^ii^	2.97
K1—B1^i^	3.465 (2)	K1—H5*B* ^ii^	2.99
K1—H01	2.53 (2)	B1—C3	1.640 (3)
K1—H5*B*	2.69	B1—C5	1.640 (3)
K1—H01^i^	2.71 (2)	B1—C1	1.640 (3)
K1—H1*A* ^i^	2.76	B1—H01	1.20 (2)
K1—H1*B* ^i^	2.75		
			
C3—B1—C5	109.90 (16)	C1—B1—H01	106.1 (10)
C3—B1—C1	112.16 (16)	K1—H01—B1	113.5 (13)
C5—B1—C1	111.40 (16)	K1—H01—K1^ii^	126.7 (9)
C3—B1—H01	107.6 (10)	H01—K1—H01^i^	104.0 (4)
C5—B1—H01	109.5 (10)		

Symmetry codes: (i) x-{\script{1\over 2}}, -y+{\script{3\over 2}}, -z+1; (ii) x+{\script{1\over 2}}, -y+{\script{3\over 2}}, -z+1; (iii) -x+{\script{1\over 2}}, -y+1, z-{\script{1\over 2}}.

**Table 2:** Experimental details

Crystal data	
Chemical formula	K^+^·C_6_H_16_B^−^
*M*_r_	138.10
Crystal system, space group	Orthorhombic, *P*2_1_2_1_2_1_
Temperature (K)	100
*a*, *b*, *c* (Å)	7.4758 (3), 7.6682 (6), 14.8010 (12)
*V* (Å^3^)	848.48 (10)
*Z*	4
Radiation type	Mo *K*α
μ (mm^−1^)	0.54
Crystal size (mm)	0.3 × 0.2 × 0.15

Data collection	
Diffractometer	Oxford Diffraction Xcalibur, Eos
Absorption correction	Multi-scan (*CrysAlis PRO*; Agilent, 2013 ▸)
*T*_min_, *T*_max_	0.976, 1.000
No. of measured, independent and observed [*I* > 2σ(*I*)] reflections	13206, 2441, 2182
*R*_int_	0.060
(sin θ/λ)_max_ (Å^−1^)	0.704

Refinement	
*R*[*F* ^2^ > 2σ(*F* ^2^)], *wR*(*F* ^2^), *S*	0.036, 0.068, 1.04
No. of reflections	2441
No. of parameters	80
H-atom treatment	H atoms treated by a mixture of independent and constrained refinement
Δρ_max_, Δρ_min_ (e Å^−3^)	0.20, −0.23
Absolute structure	Flack *x* determined using 806 quotients [(*I* ^+^)−(*I* ^−^)]/[(*I* ^+^)+(*I* ^−^)] (Parsons*et al.*, 2013 ▸)
Absolute structure parameter	−0.05 (3)

Computer programs: *CrysAlis PRO* (Agilent, 2013 ▸), *SHELXS97* (Sheldrick, 2008 ▸), *SHELXL2018/3* (Sheldrick, 2015 ▸) and *XP* (Siemens, 1994 ▸).

## supplementary crystallographic information

### Crystal data

**Table:**

K^+^·C_6_H_16_B^−^	*D*_x_ = 1.081 Mg m^−^^3^
*M**_r_* = 138.10	Mo *K*α radiation, λ = 0.71073 Å
Orthorhombic, *P*2_1_2_1_2_1_	Cell parameters from 2443 reflections
*a* = 7.4758 (3) Å	θ = 2.8–26.1°
*b* = 7.6682 (6) Å	µ = 0.54 mm^−^^1^
*c* = 14.8010 (12) Å	*T* = 100 K
*V* = 848.48 (10) Å^3^	Prism, pale yellow
*Z* = 4	0.3 × 0.2 × 0.15 mm
*F*(000) = 304	

### Data collection

**Table:**

Oxford Diffraction Xcalibur, Eos diffractometer	2441 independent reflections
Radiation source: Enhance (Mo) X-ray Source	2182 reflections with *I* > 2σ(*I*)
Graphite monochromator	*R*_int_ = 0.060
Detector resolution: 16.1419 pixels mm^-1^	θ_max_ = 30.0°, θ_min_ = 2.8°
ω scans	*h* = −10→10
Absorption correction: multi-scan (CrysAlisPro; Agilent, 2013)	*k* = −10→10
*T*_min_ = 0.976, *T*_max_ = 1.000	*l* = −20→20
13206 measured reflections	

### Refinement

**Table:**

Refinement on *F*^2^	Secondary atom site location: difference Fourier map
Least-squares matrix: full	Hydrogen site location: mixed
*R*[*F*^2^ > 2σ(*F*^2^)] = 0.036	H atoms treated by a mixture of independent and constrained refinement
*wR*(*F*^2^) = 0.068	*w* = 1/[σ^2^(*F*_o_^2^) + (0.0251*P*)^2^ + 0.0172*P*] where *P* = (*F*_o_^2^ + 2*F*_c_^2^)/3
*S* = 1.04	(Δ/σ)_max_ < 0.001
2441 reflections	Δρ_max_ = 0.20 e Å^−^^3^
80 parameters	Δρ_min_ = −0.23 e Å^−^^3^
0 restraints	Absolute structure: Flack *x* determined using 806 quotients [(*I*^+^)-(*I*^-^)]/[(*I*^+^)+(*I*^-^)] (Parsons*et al.*, 2013)
Primary atom site location: structure-invariant direct methods	Absolute structure parameter: −0.05 (3)

### Special details

**Table:**

Geometry. All esds (except the esd in the dihedral angle between two l.s. planes) are estimated using the full covariance matrix. The cell esds are taken into account individually in the estimation of esds in distances, angles and torsion angles; correlations between esds in cell parameters are only used when they are defined by crystal symmetry. An approximate (isotropic) treatment of cell esds is used for estimating esds involving l.s. planes.
Refinement. The compound is achiral and crystallizes only by chance in a chiral (Sohncke) space group.

### Fractional atomic coordinates and isotropic or equivalent isotropic displacement parameters (Å^2^)

**Table:**

	*x*	*y*	*z*	*U*_iso_*/*U*_eq_	
K1	0.22297 (6)	0.87358 (6)	0.42934 (3)	0.01798 (12)	
B1	0.2627 (3)	0.6127 (3)	0.59734 (14)	0.0140 (4)	
H01	0.368 (3)	0.680 (3)	0.5467 (14)	0.020 (6)*	
C1	0.3821 (3)	0.5161 (3)	0.67567 (13)	0.0159 (4)	
H1B	0.457483	0.426196	0.646460	0.019*	
H1A	0.463245	0.603494	0.702910	0.019*	
C2	0.2753 (3)	0.4296 (3)	0.75137 (15)	0.0260 (5)	
H2C	0.200842	0.517218	0.781547	0.039*	
H2B	0.357773	0.377763	0.795257	0.039*	
H2A	0.198637	0.338312	0.725885	0.039*	
C3	0.1433 (3)	0.4737 (3)	0.53862 (14)	0.0184 (5)	
H3B	0.053383	0.420846	0.579383	0.022*	
H3A	0.077404	0.539343	0.491626	0.022*	
C4	0.2465 (3)	0.3262 (3)	0.49238 (16)	0.0271 (5)	
H4C	0.342593	0.375726	0.455348	0.041*	
H4B	0.164952	0.259304	0.453846	0.041*	
H4A	0.298115	0.249185	0.538342	0.041*	
C5	0.1323 (3)	0.7630 (3)	0.64061 (14)	0.0150 (4)	
H5B	0.067029	0.820394	0.590574	0.018*	
H5A	0.042278	0.704783	0.679326	0.018*	
C6	0.2249 (3)	0.9042 (3)	0.69656 (16)	0.0270 (5)	
H6C	0.284544	0.850412	0.748517	0.041*	
H6B	0.135728	0.988325	0.717930	0.041*	
H6A	0.313644	0.964283	0.659107	0.041*	

### Atomic displacement parameters (Å^2^)

**Table:**

	*U*^11^	*U*^22^	*U*^33^	*U*^12^	*U*^13^	*U*^23^
K1	0.01299 (19)	0.0216 (2)	0.0194 (2)	0.00053 (18)	−0.00233 (19)	0.0028 (2)
B1	0.0099 (10)	0.0164 (10)	0.0156 (10)	0.0015 (10)	0.0008 (8)	0.0005 (8)
C1	0.0112 (10)	0.0187 (10)	0.0178 (11)	0.0003 (8)	0.0013 (8)	0.0028 (9)
C2	0.0195 (11)	0.0318 (12)	0.0267 (12)	0.0025 (10)	0.0045 (10)	0.0133 (9)
C3	0.0145 (10)	0.0199 (11)	0.0207 (11)	0.0033 (8)	−0.0003 (8)	−0.0018 (8)
C4	0.0236 (13)	0.0251 (11)	0.0326 (12)	0.0052 (9)	−0.0056 (10)	−0.0120 (9)
C5	0.0130 (10)	0.0163 (10)	0.0158 (10)	−0.0002 (8)	−0.0018 (8)	−0.0013 (8)
C6	0.0185 (10)	0.0248 (12)	0.0377 (13)	0.0016 (10)	−0.0033 (11)	−0.0127 (9)

### Geometric parameters (Å, º)

**Table:**

K1—C1^i^	3.103 (2)	B1—C1	1.640 (3)
K1—B1	3.205 (2)	B1—H01	1.20 (2)
K1—C5	3.310 (2)	C1—C2	1.527 (3)
K1—C3^ii^	3.387 (2)	C1—H1B	0.9900
K1—C5^ii^	3.396 (2)	C1—H1A	0.9900
K1—B1^i^	3.465 (2)	C2—H2C	0.9800
K1—C2^iii^	3.513 (2)	C2—H2B	0.9800
K1—C3	3.518 (2)	C2—H2A	0.9800
K1—H01	2.53 (2)	C3—C4	1.530 (3)
K1—H5B	2.69	C3—H3B	0.9900
K1—H01^i^	2.71 (2)	C3—H3A	0.9900
K1—H1A^i^	2.76	C4—H4C	0.9800
K1—H1B^i^	2.75	C4—H4B	0.9800
K1—H2B^iii^	2.83	C4—H4A	0.9800
K1—H3B^ii^	2.93	C5—C6	1.529 (3)
K1—H3A	2.93	C5—H5B	0.9900
K1—H5A^ii^	2.94	C5—H5A	0.9900
K1—H3A^ii^	2.97	C6—H6C	0.9800
K1—H5B^ii^	2.99	C6—H6B	0.9800
B1—C3	1.640 (3)	C6—H6A	0.9800
B1—C5	1.640 (3)		
			
C1^i^—K1—B1	129.41 (5)	B1—C1—H1A	108.4
C1^i^—K1—C5	112.01 (5)	K1^ii^—C1—H1A	61.0
B1—K1—C5	29.10 (5)	H1B—C1—H1A	107.5
C1^i^—K1—C3^ii^	137.56 (5)	C1—C2—K1^iv^	146.66 (14)
B1—K1—C3^ii^	91.22 (5)	C1—C2—H2C	109.5
C5—K1—C3^ii^	98.42 (5)	K1^iv^—C2—H2C	96.6
C1^i^—K1—C5^ii^	132.18 (5)	C1—C2—H2B	109.5
B1—K1—C5^ii^	87.74 (5)	K1^iv^—C2—H2B	39.9
C5—K1—C5^ii^	113.18 (5)	H2C—C2—H2B	109.5
C3^ii^—K1—C5^ii^	46.63 (5)	C1—C2—H2A	109.5
C1^i^—K1—B1^i^	28.23 (5)	K1^iv^—C2—H2A	79.5
B1—K1—B1^i^	101.50 (5)	H2C—C2—H2A	109.5
C5—K1—B1^i^	84.95 (5)	H2B—C2—H2A	109.5
C3^ii^—K1—B1^i^	157.94 (5)	C4—C3—B1	116.29 (17)
C5^ii^—K1—B1^i^	150.39 (5)	C4—C3—K1^i^	141.80 (14)
C1^i^—K1—C2^iii^	78.93 (6)	B1—C3—K1^i^	101.88 (11)
B1—K1—C2^iii^	99.69 (6)	C4—C3—K1	110.68 (14)
C5—K1—C2^iii^	122.65 (5)	B1—C3—K1	65.47 (10)
C3^ii^—K1—C2^iii^	109.29 (5)	K1^i^—C3—K1	85.41 (5)
C5^ii^—K1—C2^iii^	64.15 (5)	C4—C3—H3B	108.2
B1^i^—K1—C2^iii^	86.47 (5)	B1—C3—H3B	108.2
C1^i^—K1—C3	109.21 (5)	K1^i^—C3—H3B	55.0
B1—K1—C3	27.73 (5)	K1—C3—H3B	138.8
C5—K1—C3	46.18 (5)	C4—C3—H3A	108.2
C3^ii^—K1—C3	113.21 (5)	B1—C3—H3A	108.2
C5^ii^—K1—C3	91.37 (5)	K1^i^—C3—H3A	57.4
B1^i^—K1—C3	84.88 (5)	K1—C3—H3A	47.0
C2^iii^—K1—C3	76.61 (5)	H3B—C3—H3A	107.4
C1^i^—K1—H01	148.8 (5)	C3—C4—H4C	109.5
B1—K1—H01	20.1 (5)	C3—C4—H4B	109.5
C5—K1—H01	44.7 (5)	H4C—C4—H4B	109.5
C3^ii^—K1—H01	73.1 (5)	C3—C4—H4A	109.5
C5^ii^—K1—H01	69.0 (5)	H4C—C4—H4A	109.5
B1^i^—K1—H01	121.4 (5)	H4B—C4—H4A	109.5
C2^iii^—K1—H01	97.1 (5)	C6—C5—B1	116.11 (17)
C3—K1—H01	40.9 (5)	C6—C5—K1	103.74 (13)
C3—B1—C5	109.90 (16)	B1—C5—K1	71.90 (10)
C3—B1—C1	112.16 (16)	C6—C5—K1^i^	142.33 (14)
C5—B1—C1	111.40 (16)	B1—C5—K1^i^	101.54 (11)
C3—B1—K1	86.80 (11)	K1—C5—K1^i^	88.60 (5)
C5—B1—K1	79.00 (11)	C6—C5—H5B	108.3
C1—B1—K1	151.74 (13)	B1—C5—H5B	108.3
C3—B1—K1^ii^	119.99 (12)	K1—C5—H5B	44.0
C5—B1—K1^ii^	127.85 (13)	K1^i^—C5—H5B	57.7
C1—B1—K1^ii^	63.51 (10)	C6—C5—H5A	108.3
K1—B1—K1^ii^	89.12 (5)	B1—C5—H5A	108.3
C3—B1—H01	107.6 (10)	K1—C5—H5A	143.3
C5—B1—H01	109.5 (10)	K1^i^—C5—H5A	54.9
C1—B1—H01	106.1 (10)	H5B—C5—H5A	107.4
K1—B1—H01	46.4 (10)	C5—C6—H6C	109.5
K1^ii^—B1—H01	42.8 (10)	C5—C6—H6B	109.5
C2—C1—B1	115.49 (18)	H6C—C6—H6B	109.5
C2—C1—K1^ii^	156.25 (14)	C5—C6—H6A	109.5
B1—C1—K1^ii^	88.26 (11)	H6C—C6—H6A	109.5
C2—C1—H1B	108.4	H6B—C6—H6A	109.5
B1—C1—H1B	108.4	K1—H01—B1	113.5 (13)
K1^ii^—C1—H1B	60.2	K1—H01—K1^ii^	126.7 (9)
C2—C1—H1A	108.4	H01—K1—H01^i^	104.0 (4)
			
C3—B1—C1—C2	−66.0 (2)	K1^ii^—B1—C3—K1^i^	−166.41 (7)
C5—B1—C1—C2	57.7 (2)	C5—B1—C3—K1	77.09 (14)
K1—B1—C1—C2	164.9 (2)	C1—B1—C3—K1	−158.41 (16)
K1^ii^—B1—C1—C2	−179.55 (19)	K1^ii^—B1—C3—K1	−87.13 (10)
C3—B1—C1—K1^ii^	113.58 (14)	C3—B1—C5—C6	−179.27 (18)
C5—B1—C1—K1^ii^	−122.76 (14)	C1—B1—C5—C6	55.8 (2)
K1—B1—C1—K1^ii^	−15.5 (3)	K1—B1—C5—C6	−96.78 (17)
B1—C1—C2—K1^iv^	161.9 (2)	K1^ii^—B1—C5—C6	−16.6 (3)
K1^ii^—C1—C2—K1^iv^	−17.0 (6)	C3—B1—C5—K1	−82.48 (14)
C5—B1—C3—C4	179.06 (18)	C1—B1—C5—K1	152.57 (16)
C1—B1—C3—C4	−56.4 (2)	K1^ii^—B1—C5—K1	80.16 (12)
K1—B1—C3—C4	101.97 (17)	C3—B1—C5—K1^i^	2.19 (17)
K1^ii^—B1—C3—C4	14.8 (2)	C1—B1—C5—K1^i^	−122.76 (14)
C5—B1—C3—K1^i^	−2.19 (17)	K1—B1—C5—K1^i^	84.67 (6)
C1—B1—C3—K1^i^	122.31 (13)	K1^ii^—B1—C5—K1^i^	164.83 (9)
K1—B1—C3—K1^i^	−79.28 (7)		

Symmetry codes: (i) *x*−1/2, −*y*+3/2, −*z*+1; (ii) *x*+1/2, −*y*+3/2, −*z*+1; (iii) −*x*+1/2, −*y*+1, *z*−1/2; (iv) −*x*+1/2, −*y*+1, *z*+1/2.
